# Interfacial Microstructure and Properties of Si_3_N_4_ Ceramics/Cu/304 Stainless Steel Brazed by Ti40Zr25B0.2Cu Amorphous Solder

**DOI:** 10.3390/ma11112226

**Published:** 2018-11-09

**Authors:** Xiangping Xu, Yi Wang, Jiasheng Zou, Chunzhi Xia

**Affiliations:** School of Materials Science and Engineering, Jiangsu University of Science and Technology, Zhenjiang 212003, China; 201100003039@just.edu.cn (X.X.); m18362885806@163.com (Y.W.); cz_xia@126.com (C.X.)

**Keywords:** Si_3_N_4_ ceramics, intermediate layer, Cu interfacial structure, connection strength

## Abstract

Si_3_N_4_ ceramics and 304 stainless steel were brazed by Ti40Zr25B0.2Cu amorphous solder, and the interfacial microstructure of brazed joint Si_3_N_4_ ceramics/Ti40Zr25B0.2Cu/Cu/Ti40Zr25B0.2Cu/304 stainless steel was analyzed. The mechanical properties of the brazed joint were overtly affected by the brazing temperature and Cu foil thickness. The results revealed that the interface structure of the brazed joint might be 304 stainless steel/FeTi/Cu-Zr+Cu-Ti+Fe-Ti/Cu(s,s)/Cu-Zr+Cu-Ti+Fe-Ti/Ti-Si+Zr-Si/TiN/Si_3_N_4_ ceramics. The four-point bending strength of the brazed joint decreased sharply as the brazing temperature increased and reached a maximum of 76 MPa at 1223 K. Furthermore, as the Cu foil thickness was increased from 500 μm to 1000 μm, the joint strength rose to 90 MPa at 1223 K.

## 1. Introduction

Engineered ceramics have many excellent physical and chemical properties such as high hardness and strength, and resistance to high temperature, corrosion, and abrasion. These superior properties promote the wide use of engineered ceramics in aerospace, optics, energy, and the machinery field [1,2]. However, some deficiencies of engineering ceramics themselves, such as the high brittleness, the difficulty in processing, and the low plasticity and toughness, have limited the application in these fields [3,4]. The connection of ceramic and metal to obtain high-quality joints will achieve the optimization of the respective properties of ceramics and metals and combine the advantages of high hardness, high strength, and easy processing of metals [5]. Therefore, the application scope of engineered ceramics can be expanded [6,7], and reliable joints of ceramics and metals should be assessed for successful manufacturing.

Brazing and diffusion bonding is the most common way to connect ceramics and metals. Although a large number of studies have been carried out on conventional brazing ceramics/metals at home and abroad, the bonding strength is generally not high in order to further improve the mechanical properties of the ceramic/metal brazed joints and give full play to the advantages of the two materials, and to realize smooth connection of ceramic and metal materials with greater differences in thermal physical properties. In recent years, the craze of high-quality ceramic/metal connection has been achieved by adding intermediate layers, using composite brazing fillers and amorphous brazing fillers. Katsuak [8] and others brazed Si_3_N_4_ ceramic/45 steel by adding Invar alloy as a stress buffer layer, mainly through the low thermal physical properties of the intermediate layer to neutralize the larger thermal physical properties difference between the two base materials, thus obtaining the joints with higher strength. Xian [9] and others tried to braze Si_3_N_4_ ceramic/40Cr steel with different composite intermediate layers. The composite intermediate layers were designed as Cu/Nb, Cu/Ta, and Cu/Mo. The research shows that the use of composite intermediate layers can improve the stress distribution of brazed joints and increase the bending strength of joints. Zou [10] and others used a melt-spinning method to synthesize an amorphous active solder foil having a composition of Ti40Zr25Ni15Cu20 in a roll forging machine in an argon atmosphere. The silicon nitride ceramic was brazed with a synthetic Ti40Zr25Ni15Cu20 amorphous foil. The effects of brazing temperature and time on joint strength and interface microstructure were investigated. Chang [11] and others studied contributions of a soft intermediate layer of Cu when Si_3_N_4_ ceramics/316 stainless steel was connected to improve residual stress distribution. Cao Jian [12] successfully realized the brazing connection of SiC/Cu/304 stainless-steel using the conventional Ag-Cu-Ti solder, which mainly discussed the effect of the stress buffer layer Cu on the microstructure and bending strength of the joint [13,14]. Takahiro [15] and others used a Fe-based filler and realized the ceramic and stainless-steel vacuum brazing connection at 1323 K temperature. The highest shear strength of the joint was 57 MPa, and the brittle phase was easily generated by the P element in high reaction temperatures. Xu [16] and others used an Ag-Cu-Ti filler and a copper middle layer, which realized Si_3_N_4_ ceramics brazed with 304 stainless-steel at 1153 K temperature in a vacuum environment. The bending strength of four points at room temperature was 57 MPa when the thickness of the copper middle layer was 500 μm. The result showed that the connection between Si_3_N_4_ ceramics and 304 stainless steel can be realized at a lower temperature. However, the strength performance of the joint was low. Zou [17] and others designed a series of different compositions of titanium-nickel-copper brazing filler metal to study the influence of alloying elements B, silicon and zirconium on the amorphous forming ability and properties of brazing filler metal. The results show that trace amounts of boron and silicon can significantly improve the wettability of titanium-nickel-copper brazing filler metal to silicon nitride ceramics. Xia [18] and others studied the thermal fatigue properties of W-Cu/1Cr18Ni9 steel brazed joints containing Ag-Cu filler metal. Under the external bending load and internal fatigue damage, the fracture characteristics of the W-Cu/Ag-Cu1Cr18Ni9 joint changed from the ductile fracture of the original joint to the mixed ductile-brittle fracture after 200 cycles of fatigue. The ductile fracture is located on the Ag-based solid solution of the brazed joint, and the W-Cu composite material undergoes brittle fracture.

In order to obtain high quality ceramic/metal brazed joints, it is necessary to adjust the brazing process to ensure the good wettability of the brazing alloy on the ceramic base metal, which is the key to a smooth reaction. In this paper, the brazed joint of Si_3_N_4_ ceramics/304 stainless steel was realized by a Ti40Zr25B0.2Cu amorphous solder, with Cu as the middle layer. The amorphous filler was able to keep its uniformity in the liquid state. When the filler metal was melted, there was no grain and eutectic phase formation. The composition of the brazed joint was more stable than crystalline filler.

## 2. Materials and Methods 

The base materials were Si_3_N_4_ ceramics and 304 stainless steel. Si_3_N_4_ ceramics were provided by Qinghua University (Beijing, China), and sintered by hot pressing. The size was 19 mm × 19 mm × 8 mm. They were made of commonly used Si_3_N_4_ ceramics, a small amount of conductive TiC, and other components, and had excellent properties, such as small high-temperature creep, corrosion resistance, high temperature strength, wear resistance, and good thermal shock resistance. The main performance parameters are shown in Table 1. The 304 stainless steel was 19 mm × 19 mm × 8 mm. Ti40Zr25B0.2Cu of amorphous solder was made in the laboratory. The brazing filler metal was smelted in a WK-type high-vacuum Ar atmosphere-protected non-consumable electric arc furnace, and then a crystalline bulk solder of the same composition was prepared using a HVDS-II high-vacuum single-roller belt staking machine (Shenyang, China). The raw materials for preparing the Ti40Zr25B0.2Cu amorphous solder foil strip were Ti, CuB alloy, Cu, and Zr. The purity of Cu and Ti was higher than 99.9%, the purity of Zr was higher, up to 99.99%, and the content of B in CuB alloy was 13.45%. The ratio of each raw material (wt.%) required for preparing the Ti40Zr25B0.2Cu amorphous brazing filler metal was: Ti:Zr:B:Cu = 40:25:0.2:balance. The middle layer of Cu consisted of pure copper.

Before brazing, the surface of 304 stainless steel and Si_3_N_4_ ceramics were polished by sandpaper and glass, respectively, to ensure that the surface to be welded was smooth, but there was a certain roughness. Then, the specimens were cleaned using alcohol and submitted to ultrasonic cleaning for about 15 min with acetone to remove the surface oil and dirt. The oxide layer of Ti40Zr25B0.2Cu amorphous brazing and the surface of the middle Cu layer were removed with sandpaper preferentially [15] and ultrasonically cleaned in acetone. The Si_3_N_4_/Ti40Zr25B0.2Cu/Cu/Ti40Zr25B0.2Cu/304 stainless steel was ready for brazing. Brazing was carried out in a KJL-2 multi-functional vacuum furnace (Beijing, China). After the vacuum degree was pumped to 8.0 × 10^−3^ Pa, the program to heat up was started. The temperature was raised at the rate of 10 K/min to 1073 K first, then insulated for 30 min. The temperature continued to increase to the brazing temperature (T) at 15 K/min. The temperature was kept high for a period of time (t), reduced to 300 K at the rate of 4 K/min, and finally cooled to room temperature.

The brazed specimens were cut perpendicular to the interface direction and prepared as metallographic specimens. The morphology of the connection interface area was observed and analyzed on a JEOL-6480 scanning electron microscope (Shimadzu, Kyoto, Japan). Elemental distribution of the connection interface area was observed and analyzed by energy dispersive spectroscopy (MODEL7573) (Shimadzu, Kyoto, Japan). The sample was processed into a bending specimen and the four-point bending strength at the room temperature of the joint was measured on a CMT5205 electronic universal testing machine (Zhuhai, China). The displacement rate of the indenter was not more than 0.5 mm/min. The average temperature of the specimen was measured with the average of six specimens.

## 3. Results

### 3.1. Analysis of Interface Structure

**Figure 1:**
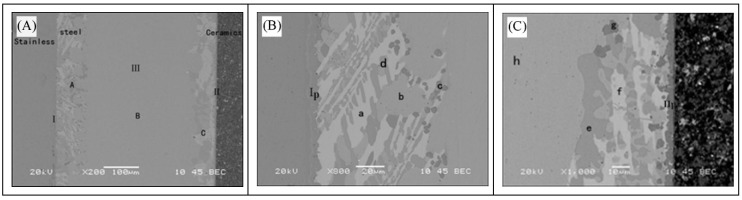
(**A**) Interfacial structure of 304/Ti40Zr25B0.2Cu/Cu/ Ti40Zr25B0.2Cu/ Si_3_N_4_; (**B**) stainless steel side; (**C**) ceramic side.

Figure 1 represents backscattered electrons of the Si_3_N_4_/Ti40Zr25B0.2Cu/Cu/Ti40Zr25B0.2Cu/304 brazed joint at a brazing temperature of 1223 K. This temperature was kept for 30 min. The pressure was 0.027 MPa for an intermediate Cu layer thickness of 500 μm. Figure 1B shows a high-magnification backscattered electron phase in the stainless steel side interface reaction layer of the brazed joint. Figure 1C depicts a high-magnification backscattered electron phase in the ceramic side interface reaction layer. As shown in Figure 1A, no obvious defects were found throughout the brazing seam zone. The connection interface of ceramics, solder, and stainless steel was clear. These results indicated that the use of Ti40Zr25B0.2Cu amorphous brazing and a Cu intermediate layer achieved good connections of ceramics and stainless steel. From left to right in the figure, the entire joint interface of stainless steel to ceramic can be divided into three regions and two interfaces. Three regions, in turn from left to right, were stainless steel, brazing seam center, and ceramics. The two interfaces were the side reaction layers of stainless steel and ceramics, respectively. 

To facilitate subsequent analysis, the side reaction layer of 304 stainless steel was called I. The reaction layer of Si_3_N_4_ side was termed II. The brazing seam center was named III. The brazing seam center III occupied most of the middle region of the brazed joint. The characteristic microstructure could be divided into the three different areas A, B, and C. The reaction layer I was the reaction layer of 304 stainless-steel/Ti40Zr25B0.2Cu amorphous brazing. It was mainly produced by diffusion and reaction of active Ti in stainless steel and solder, and along the stainless steel matrix. The reaction layer had no obvious defects and was serrated, and the brazed joint had good quality. The reaction layer II was a continuous reaction layer of the Si_3_N_4_ side, as brazing transition connection interface of ceramics and Ti40Zr25B0.2Cu amorphous. This was the key to successful connection.

**Figure 2:**
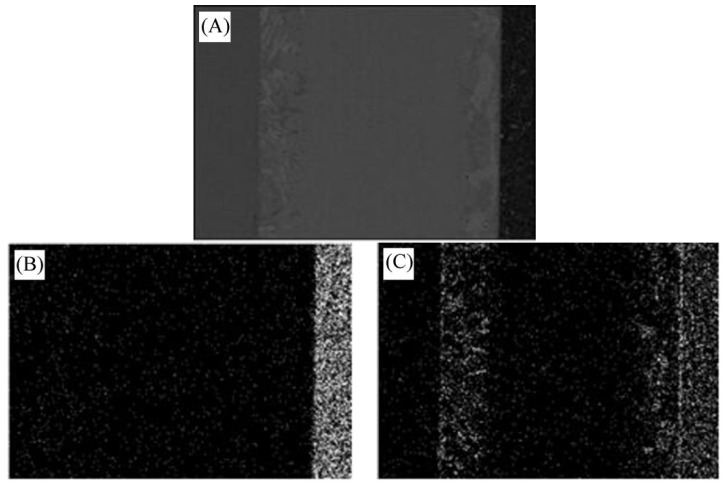

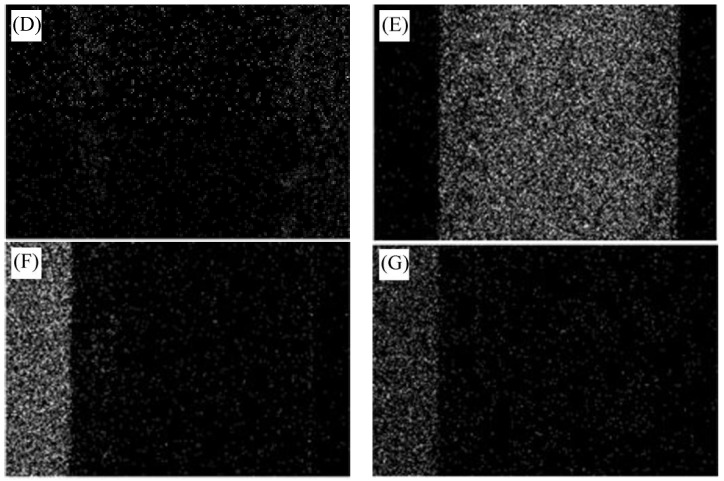
Main elements of the joint surface scanning interface: (**A**) Surface scanning position; (**B**) distribution of Si element; (**C**) distribution of Ti element; (**D**) distribution of Zr element; (**E**) distribution of Cu element; (**F**) distribution of Fe element; (**G**) distribution of Cr element.

In order to further analyze the composition of each reaction phase in the interface of 304 stainless steel/Ti40Zr25B0.2Cu/Cu/Ti40Zr25B0.2Cu/Si_3_N_4_ ceramic brazing joints, the surface scanning of several main elements in the brazed joint area was analyzed, as shown in Figure 2. The stainless steel side was mainly covered with Fe and Cr elements. The reaction layer I mainly consisted of the linear distribution of Ti elements. The brazing seam was mainly composed of Cu and Zr elements. Reaction layer II was also mainly composed of Ti elements with uniform linear distribution, but it was brighter than the distribution of Ti in the reaction layer I. The ceramic side was mainly composed of Si.

**Figure 3:**
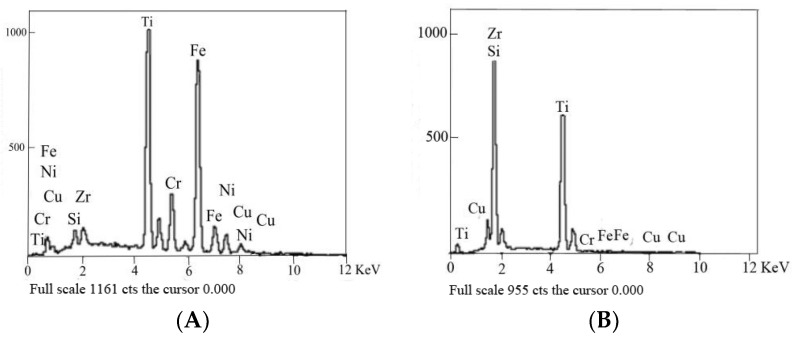
Main element points scan results of stainless steel/Ti40Zr25B0.2Cu/Cu/Ti40Zr25B0.2Cu/ceramics: (**A**) Point I; (**B**) point II.

To determine the reaction product of 304 stainless steel/Ti40Zr25B0.2Cu/Cu/Ti40Zr25B0.2Cu/Si_3_N_4_ ceramic brazed joints in each area, different location points were selected for scanning. The compositions of Figure 1B,C feature points were analyzed by energy dispersive spectroscopy. Table 2 shows EDS components for each feature point analyzed (Unit: mass fraction, wt.%). The spectrum lines of points I and II are shown in Figure 3.

Scan point I was located at the stainless-steel/solder connection interface and mainly contained Fe and Ti elements. The corresponding precipitated phase diagram was an Fe-Ti metal compound according to the Fe-Ti binary phase diagram. Fe to Ti atomic ratio was approximately 4:3. The product phase was identified as FeTi. The formation of the phase depended mainly on the diffusion of Fe from the stainless-steel matrix to the Ti40Zr25B0.2Cu amorphous solder and the diffusion of active element Ti from the amorphous solder to the base metal. 

Scan point a was located in the stainless-steel side and strip white phase, close to the reaction layer of stainless-steel. The main elements were Cu and Zr. With the increase of temperature during brazing, the melting of amorphous solder and the diffusion of elements in brazing filler metals were intensified, especially the active elements Ti and Zr. Active element Zr of the brazing material and the intermediate Cu layer were combined to generate the Cu-Zr compound. The span of the white phase in the figure could to some extent reflect the degree of diffusion of active Zr to the center of the braze under the brazing condition. The corresponding precipitated phase diagram was CuZr2 according to the Cu-Zr binary phase diagram. Scan point b was a light gray matrix, mainly composed of small amounts of Cu, Fe, and Ti. This phase dissolved a small amount of Fe in stainless steel and Cu-based solid solution of Ti in solder. Scan point c in the diagram was presented in dark gray and had Fe-Ti and Cu-Ti compounds. With the increase of brazing temperature, the stainless-steel base material was continuously dissolved into the solder, free Fe was present on the interface, and the diffusion ability of Fe was very strong so that the Fe-Ti phase was formed in the direction of the center of the brazing seam and the deep grey granular phase was formed together with the Cu-Ti compound. Scan point d was the strip gray phase and the main phase component was the Cu-Ti compound. The corresponding precipitated phase was CuTi and CuTi2 compounds according to the Cu-Ti binary phase diagram. Scan point e represented the gray massive microstructure and was located at the scanning spot of the brazing seam center. The corresponding precipitated phase diagram was the same as the CuTi and CuTi2 compounds according to the Cu-Ti binary phase diagram. The gray phase of areas A and C was CuTi and possibility CuTi2.

White phase scan spot f of the ceramics side was similar to the white phase of stainless steel. The main phase was Cu-Zr and the corresponding precipitated phase was the CuZr2 compound according to the Cu-Zr binary phase diagram. Scan spot g was located at dark gray particles of the ceramic side. The main distribution involved Ti, Cu, and Fe element. In accordance with the analysis of 304 stainless steel, the dark gray particles of the side may be the same as Fe-Ti and Cu-Ti compounds. The main component of scan point h at brazing seam center was Cu and it dissolved with small amounts of Cu, Ti, and Cr elements. The light gray uniform phase of the central region was the intermediate Cu layer, which was solubilized but not consumed by solder. 

Scan spot I was located at the reaction layer of the ceramics side of the ceramics/solder transition interface. In addition to N element, which was not detected, the main elements were Si, it, and Zr. Si_3_N_4_ and the reaction of active Ti and Zr element were key to successful completion of the brazing connection. Though Ti and Zr were the same active elements, the activity of Zr was not as good as that of the Ti element. Ti was therefore superior to Zr. The Ti element participated in interfacial reaction and generated the TiN layer. This TiN layer played a role in multiple great thermo-physical properties of welded joints and improved the performance of the joint. Meanwhile, free Si displaced from the reaction between Si_3_N_4_ and Ti was diffused from the Si_3_N_4_ ceramic base material to the amorphous solder due to the concentration gradient and the active Ti in the amorphous solder was continuously diffused to the base material of Si_3_N_4_ ceramic. Si reacted with Ti and Zr enriched near the TiN interface reaction layer to form Ti-Si and Zr-Si compounds near the TiN reaction layer. 

In summary, the main phase of the 304 stainless-steel side were the FeTi and Fe2Ti compounds. The Si_3_N_4_ ceramics side contained TiN, Ti-Si, and Zr-Si. The center of the brazing seam was CuZr2, Fe-Ti, Cu-Ti, and Cu-based solid solution. 

### 3.2. Performance of Brazed Joints

The mechanical properties of brazed joints were affected by many factors, such as operating temperature, load properties, the joint interface, brazing environment, composition of base metal, and residual stress. Among them, the influence of the interface structure on the mechanical properties of the joint was an essential factor that directly determined the performance of the joint.

From the foregoing, it can be seen that the brazing temperature and the thickness of the Cu foil in the intermediate layer are important factors affecting the interface structure of the joint. Therefore, the mechanical properties of joints with ceramics and stainless steel were mainly influenced by brazing temperature and thickness of the intermediate Cu foil layer. The results of four-point bending strength are shown in Table 3.

### 3.3. Effects of Brazing Temperature on Mechanical Properties

**Figure 4:**
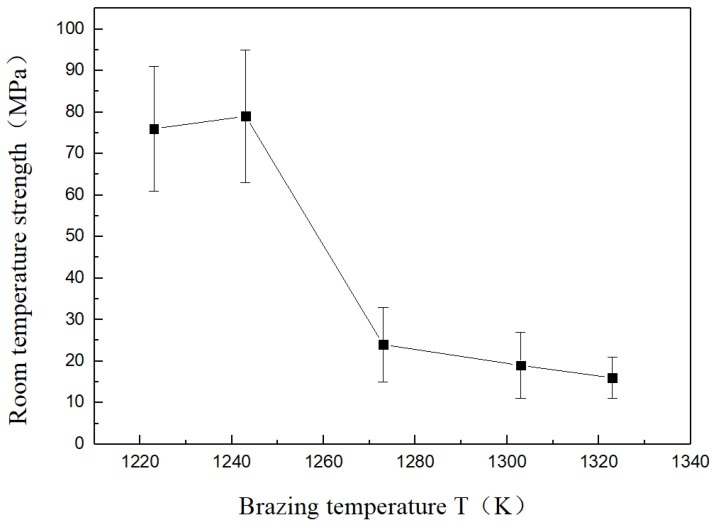
Brazing temperature effects on joint strength at room temperature (500 μm).

In order to study the influence of brazing temperature on the strength of a 304 stainless steel/Ti40Zr25B0.2Cu/Cu/Ti40Zr25B0.2Cu/Si_3_N_4_ joint at room temperature, intermediate thickness, holding time, and other process parameters were fixed and only brazing temperature was changed. The influence of the brazing temperature on the strength of the joint at room temperature is shown in Figure 4.

When the brazing temperature was 1223 K, with the interaction between the amorphous brazing alloy and the base metal, the interfacial layer on both sides of the ceramic/stainless steel was clear. In particular, the ceramic side interface reaction layer was continuous and straight, mainly due to the fine grain TiN layer formed by interfacial reaction, which had good compactness and excellent connection. The four-point bending strength was 76 MPa. When the brazing temperature increased to 1243 K, the increase of brazing temperature made the diffusion of atoms more obvious and the thickness of the reaction layer increased. The four-point bending strength slightly increased to 79 MPa, so the connection was excellent with the brazing temperature increased to 1243 K. When the brazing temperature increased to 1273 K, the reaction between atoms was intensified with the increased brazing temperature. Atomic diffusion was more obvious and the reaction layer thickness increased constantly. Meanwhile, Ti-Si, Zr-Si, and Cu-Zr compounds of the ceramic side had a connected interface with the Cu-Zr compound. The ceramic side near the brazing seam center also increased. Even micro-cracks were found in the microstructure of Cu-Zr. The presence of microcracks can easily induce cracks to initiate and quickly propagate to the ceramic. Eventually, the ceramic near the ceramic interface underwent brittle fracture, so the room temperature strength was drastically reduced to 24 MPa when the brazing temperature reached 1303 K. The middle layer of copper continued to be consumed. Further deterioration of joint performance got worse with the growth of the Ti-Si and Cu-Zr compounds of the ceramics. With the Cu layer completely consumed, when the brazing temperature reached 1323 K the brittle intermetallic compounds Cu-Zr, Cu-Ti, and other discontinuities covered the entire solder joints. Brittle Ti-Si and Zr-Si intermetallic compounds continued to grow. The performance of the joint was deteriorated further under the effect of thermal stress, and joint strength was lower.

### 3.4. Effects of Cu Foil Thickness on Mechanical Properties

**Figure 5:**
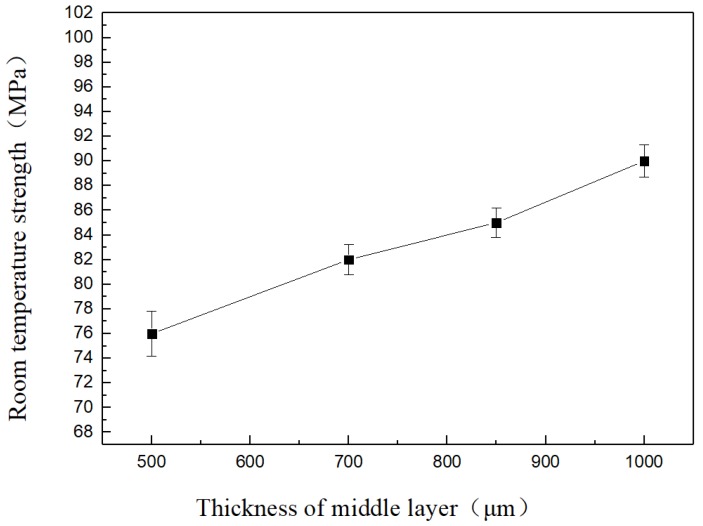
Thickness of middle layer effects on joint strength at room temperature (1223 K).

As can be seen from Figure 5, when the brazing temperature was 1223 K the strength of solder joints at room temperature showed a significant upward trend with increasing thickness of the intermediate Cu foil layer. The intermediate Cu foil layer was 1000 μm at a brazing temperature of 1223 K and reached a maximum strength of 90 MPa at room temperature. This was mainly due to the lower yield strength of the soft intermediate layer Cu, which eased the residual stress of the joint through self-plastic deformation. The thicker the Cu foil, the more obvious the effect to improve the stress distribution of the joint and improve the mechanical properties of the joint.

### 3.5. Fracture Path of Joint

**Figure 6:**
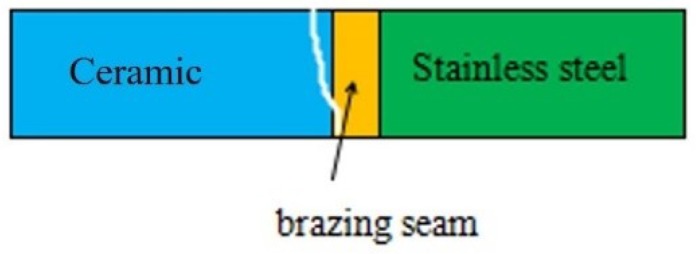
The fracture of 304/Ti40Zr25B0.2Cu/Cu/Ti40Zr25B0.2Cu/Si_3_N_4_ joint.

**Figure 7:**
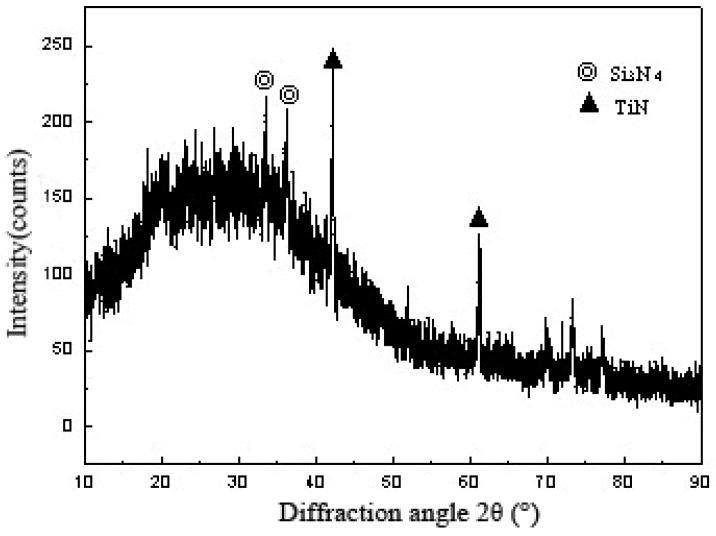
XRD result of fracture surface in the ceramic side.

**Figure 8:**
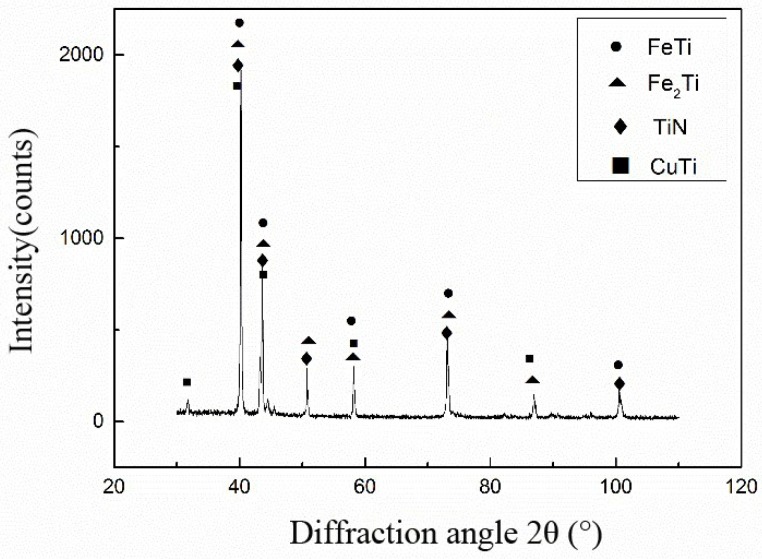
XRD result of fracture section.

Figure 6 shows the facture schematic diagram of a Si_3_N_4_/Ti40Zr25B0.2Cu/Cu/Ti40Zr25B0.2Cu/304 stainless steel welding joint. The four-point bending fracture position was clearly observed at the surface of the ceramic material. Because of higher residual stress in the welding joint, the peak stress appeared at the base of the side of the brazing seam. Meanwhile, the plasticity of ceramic materials was poor, which led to the weakened position of the welding joint. If the brazing joints had cracks, non-fusion gaps, and other defects, the crack expanded rapidly to the interior of the ceramic under the action of the thermal stress of the joint. In the end, brittle fracture occurred at the surface of the ceramic material. Figure 7 shows the XRD analysis result of the ceramic side section of the Si_3_N_4_/Ti40Zr25B0.2Cu/Cu/Ti40Zr25B0.2Cu/304 stainless-steel welding joint, which can be used to verify the existence of the brazing joint interface TiN. Figure 8 shows the XRD analysis result of the fracture section of the Si_3_N_4_/Ti40Zr25B0.2Cu/Cu/Ti40Zr25B0.2Cu/304 stainless-steel welding joint, which can be used to verify the existence of the brazing joint interface FeTi, Fe2Ti, TiN, and CuTi.

## 4. Conclusions

(1) 304/Ti40Zr25B0.2Cu/Cu/Ti40Zr25B0.2Cu/Si_3_N_4_ ceramics were brazed by Ti40Zr25B0.2Cu amorphous solder. A good brazed joint was obtained and joint interface was continuous and dense.
(2) The interface structure of the 304/Ti40Zr25B0.2Cu/Cu/Ti40Zr25B0.2Cu/Si_3_N_4_ ceramics joint might be 304/FeTi/Cu-Zr+Cu-Ti+Fe-Ti/Cu(s,s)/Cu-Zr+Cu-Ti+Fe-Ti/Ti-Si+Zr-Si/TiN/Si_3_N_4_ ceramics from left to right.
(3) Joint strength at room temperature rapidly decreased with increasing brazing temperature. The performance of welded joints was improved significantly with increasing Cu foil layer thickness. With a brazing temperature of 1223 K and an intermediate Cu foil layer of welded joint of 1000 μm, the strength at room temperature reached a maximum of 90 MPa.

## Figures and Tables

**Table 1:** Main performance parameters of Si_3_N_4_ ceramics.

Project of Performance	Hot-Pressing Si_3_N_4_ Ceramics
Density (g/cm^3^)	3.25–3.35
Hardness (HRA)	92–94
Modulus of elasticity (GPa)	304–330
Thermal expansion coefficient (10^−6^/K)	3.2–3.5
Coefficient of thermal conductivity (J/(cm·s·K))	0.155–0.293
△H0298 (KJ/mol)	−749

**Table 2:** EDS result of the feature points in Figure 1.

Point	Si	Ti	Zr	Cu	Fe	Cr	Ni	Possible Phase
I_p_	1.25	28.86	3.01	3.73	46.51	9.58	7.06	FeTi
II_p_	34.68	54.42	9.54	0.72	0.37	0.27	-	TiN, Ti-Si
a	-	1.95	22.88	70.17	0.86	0.24	3.90	CuZr2
b	-	0.92	-	98.96	0.12	-	-	Cu(s,s)
c	0.68	45	2.84	12.68	24.4	5.53	8.88	Fe-Ti, Cu-Ti
d	-	26.67	2.42	48.14	2.09	-	-	Cu-Ti
e	0.04	32.56	1.18	64.44	1.62	0.18	-	Cu-Ti
f	0.02	6.04	21.77	71.65	0.52	-	-	CuZr2
g	2.17	60.66	2.15	21.63	12.06	1.33	-	Fe-Ti, Cu-Ti
h	0.06	0.06	-	99.78	-	0.09	-	Cu(s,s)

**Table 3:** Strength of brazed joints at room temperature.

Brazing Temperature T (K)	Thickness of Cu Foil (μm)	Room-Temperature Strength (MPa)
1223	500	76
	700	82
	850	85
	1000	90
1243	500	79
1273	500	24
1303	500	19
1323	500	16
